# “Concerns” about medical students’ adverse behaviour and attitude: an audit of practice at Nottingham, with mapping to GMC guidance

**DOI:** 10.1186/1472-6920-14-196

**Published:** 2014-09-20

**Authors:** Janet Yates

**Affiliations:** Research Fellow, Medical Education Centre, School of Medicine, University of Nottingham Medical School, Nottingham, NG7 2UH UK

**Keywords:** Medical students, Unprofessional behaviour, Audit, Fitness-to-practise, GMC guidance

## Abstract

**Background:**

The development and maintenance of students’ professional behaviour and attitude is of increasing importance in medical education. Unprofessional behaviour in doctors has the potential to jeopardise patient safety, compromise working relationships, and cause disruption and distress. The General Medical Council issues guidance to medical schools and students describing the standards that should be attained.

Nottingham University medical school introduced a ‘Concerns’ form in 2009, to create a standardised, transparent and defensible means of recording and handling complaints about adverse attitudes or behaviours. This paper reports an audit of the system over the first three years.

**Methods:**

The routinely-held database was enhanced with further detail collected from relevant student records. The data were explored in terms of the types of complaint, students who were reported, the people who reported them, and the actions taken afterwards. The data were also mapped to the current GMC guidance.

**Results:**

189 valid forms were generated, relating to 143 students. The form was used by a wide variety of people, including clinical and non-clinical teachers, administrators, Hall Wardens, and fellow students. The concerns ranged from infringements of regulations to serious fitness to practise issues. Most were dealt with by faculty or pastoral care staff but some required escalation to formal hearings. The complaints were mapped successfully to GMC documentation, with the highest proportions relating to the GMC categories ‘Good Clinical Care’ and ‘Working with Colleagues’.

Male and ethnic minority students appeared to be more likely to have a Concern raised, but this is a tentative conclusion that requires a larger sample. Undergraduate (as opposed to Graduate Entry) students may also be at greater risk.

**Conclusions:**

A simple form, freely available, but designed to prevent frivolous or malicious use, has provided valuable data on unprofessional behaviour and the responses elicited. Some parts of the form require improvements, and these are underway to provide more efficient use, audit and review in future.

**Electronic supplementary material:**

The online version of this article (doi:10.1186/1472-6920-14-196) contains supplementary material, which is available to authorized users.

## Background

The issue of professional behaviour in medical students is a current ‘hot topic’ in medical education. As professionalism in doctors comes increasingly under the spotlight, so does the need to instil the right behaviours and attitudes in students, and to monitor adverse incidents. Professional misconduct in medical students has been shown be a risk factor for subsequent disciplinary action in clinical practice [1, 2]. Subsequently, Teherani *et al.* categorised unsatisfactory behaviours demonstrated by students undertaking clinical performance examinations [3]. These were grouped, in decreasing order of frequency, as a diminished capacity for self-improvement, impaired relationships with patients, irresponsibility, poor initiative, and unprofessional behaviour associated with anxiety. Studies from Australia [4] and the Netherlands [5] support the conclusion that there is international concern over the occurrence, identification, and remediation of unsatisfactory behaviours.

In the UK, guidance for both medical schools and their students regarding professional behaviours comes from the General Medical Council (GMC). Key documents include *Tomorrow’s doctors*, which sets out the skills, knowledge and attitudes that medical students must acquire before graduation [6], and *Medical students: professional values and fitness to practice* [7]. The latter publication explains students’ obligations in developing and demonstrating professional behaviour, and how lapses may be investigated and handled through fitness to practise procedures.

When considering the occurrence of unprofessional behaviour in students, one of the first questions to ask is how to create a system for recording and handling complaints in a transparent, reliable, practical and effective way. At Nottingham we have developed a ‘Concerns’ form, which has been in use since 2009. It provides a standardised structure by which anyone, whether a teacher, fellow student, member of the public or medical school administrator, can document a genuine complaint about a student’s behaviour or attitude. The person making the complaint (described here as the ‘originator’) must sign and date their report and be prepared, if necessary, to be identified in any future proceedings. This prevents misuse of the form. All forms are logged and processed by a dedicated member of staff, using an algorithm, and are reviewed by a senior member of staff (normally a Senior Tutor or Clinical Sub-Dean) before any further action is taken. This action will depend on the severity of the complaint and the nature of the evidence. For the most minor issues, the student in question may simply be sent a warning email; more serious events may merit a formal interview; and the most serious may be escalated towards a possible Fitness to Practise hearing. A copy of the Concerns form (with related office procedure, as used in 2012), is included as an additional file to this paper [Additional file 1]. It provided four potential areas of concern: 1) Student unhappy/withdrawn/has health problems; 2) Inappropriate attitudes or behaviours; 3) Serious misconduct (eg criminal conviction or caution/drug or alcohol misuse/aggressive or threatening behaviour); and 4) Other. The form provided examples of some behaviours and incidents that were appropriate for referral.

Although the use of the forms is kept under continuous review, the layout has been relatively unchanged since its inception. The aims of this study were to see 1) if it was ‘fit for purpose’, and 2) how closely it aligned with current recommendations for student behaviour [7]. We conducted an in-depth audit, to investigate which complaints predominated, what variation there might be in the types of people making the complaints at different stages in the course, and the ultimate outcomes. As part of this process we mapped whether the concerns aligned with the GMC’s guidance.

## Methods

A copy of the active database was used as the basis for investigations, and restricted to entries between October 2009 and September 2012 inclusive. A detailed analysis was then planned, requiring some enhancements to the database. For example, complaints had been logged under the actual name of the originator, who also wrote their job title or status at the time, such as clinical lecturer or fellow student. These data were replaced in the audit database by a single anonymised code for their position or status (options listed below under Results). The actual complaint had been recorded under the four headings used in the form, ie Health, Attitude and Behaviour, Misconduct, or Other, but only a minimal amount of information had been added to the routine database. In order to increase the available information, an additional field was added to the audit database, the original form traced, and further details written to describe more fully the event or incident causing the complaint to be raised.

In addition, basic demographics of all students registered on the medical course during this time period were obtained from the Central Services department of the University. This enabled some simple comparisons to be made between the ‘errant’ students and the rest of their peers. The database was then anonymised.

Data were analysed in Access and IBM SPSS v19.

### Ethical approval

The Chair of the University of Nottingham Medical School Research Ethics Committee reviewed the proposal. Formal ethical approval was not needed for this anonymised audit of routinely-collected data.

## Results

### Overall student intake

The students listed in the database had enrolled on the course during the seven academic years 2005–6 to 2011–12 inclusive. The total intake over this period was 2436, of which 1734 (71%) had been enrolled on the standard 5-year Undergraduate (UG) course, and 638 (26%) on the 4-year Graduate Entry Medicine (GEM) course. The remaining 64 (3%) were on Nottingham’s dedicated preclinical course for Thai students.

Table 1 summarises the intake for the UG and GEM courses over this period, with numbers and percentages for sex and ethnic origin. It is clear that the GEM course admits a higher proportion of male and White students.

**Table 1:** **Summary of basic demographics of intake over seven academic years, 2005–6 to 2011–2012**

Sex and ethnic origin	Admissions for 5-year UG course	Admissions for 4-year GEM course	Admissions for special (Thai) pre-clinical course	Combined intake
Total intake n (row%)	1734 (71)	638 (26)	64 (3)	2436
Total n (column%) male	661 (38)	377 (59)	27 (42)	1065 (44)
Ethnicity				
Total n (column%) White	1135 (65)	500(78)	0	1635 (67)
Total n (column%) non-White	511 (30)	110(17)	64 (100)	685 (28)
Total n (column%) undeclared ethnicity	88 (5)	28 (5)	0	116 (5)

### The concerns database

Concerns forms had been raised on 235 occasions during the three academic years between September 2009 and 2012. However, 42 of these forms were marked ‘invalid’ and deleted from the database, because they all referred to a single incident of these students not attending part of a lecture, with a misunderstanding involved. In addition, a further four forms were discarded as being inapplicable: one where the originator had been informed that a Concerns form was inappropriate; one for a student who had missed teaching due to close family illness but had performed adequately and been signed off; one for a student whose CRB check had shown a traffic offence which had no implications for fitness to practise; and one for a student with poor attendance but who had already sought help and support. This left 189 valid forms. There were 22 students with more than one Concern form logged in their names; 13 had two forms, six had three, two had four and one student 16, generating 68 forms between them. The remaining 121 forms were for individual students, ie 143 students in total.

Of these 143 individuals, 88 were male and 55 female, ie ~62% males. In terms of ethnic origin, 76 (53%) of these students were White, 54 (38%) non-White, and 13 (9%) were of unknown ethnic group. There is evidently an excess of males and students of non-White ethnicity within those with Concerns logged, compared to the intake shown in Table 1. This difference was highly significant (p < 0.001) in both instances (for males vs females, χ^2^ = 53.3, Odds Ratio 3.1, 95% Confidence Interval 2.26 to 4.25; for non-White vs White, excluding the unknown group, χ^2^ = 322.4, Odds Ratio 3.61, 95% Confidence Interval 3.13 to 4.16).

The course codes of these students were also examined. 113/143 (79%) were UG students, very slightly above their overall representation of 71% in the student body (see Table 1). There were 24 GEM students, 17%, lower than their overall proportion of 26%. Five (3.5%) were on the dedicated course for Thai students. The remaining one was on the dedicated clinical course for Malaysian students and would not have featured on the original admission lists. GEM students were statistically less likely to have concerns raised (GEM vs UG, χ^2^ = 5.87, OR 0.58, 95% CI 0.37 to 0.91, p 0.015).

All the remaining analysis and Tables below relate to individual forms rather than individual students, so numbers will be slightly skewed by those students with multiple forms.

### Referrals by academic year

At Nottingham, students on the 5-year undergraduate (UG) course spend four semesters on largely pre-clinical studies, followed by their ‘Honours’ course in Semester 5, which includes a research project as well as taught modules. Students on the 4-year Graduate Entry Medicine (GEM) course spend three semesters on an accelerated pre-clinical course. The two groups are then combined for the five clinically-based semesters.

Table 2 provides details of the referrals by academic year. There was considerable variation in numbers/year and the spread across the course. It appeared that the last two years of the combined course generated the biggest proportions of complaints (90/189, 48%), followed by the early UG course (52/189, 28%).

**Table 2:** **Referrals by academic year (by number of forms)**

Academic year of referral *	Year 1	Year 2	Hons year 3	GEM 1	GEM 2	CP1	CP2	CP3	Total
2009-2010	16	8	11	4	7	6	13	14	79
2010-2011	7	11	2	2	0	4	7	7	40
2011-2012	1	9	5	0	1	5	29	20	70
Totals	24	28	18	6	8	15	49	41	189

* In this Table, Year 1, Year 2 and Hons Year 3 refer to the early undergraduate course; GEM 1 and GEM 2 refer to the early graduate entry course; and CP (Clinical Practice) 1, 2 and 3 to the 5-semester shared clinical course. CP1 occupies only 1 semester, CP2 & 3 are two semesters each.

### Sex and ethnic origin of referrals

In all remaining Tables, course structure has been condensed into UG pre-clinical, GEM pre-clinical, and combined clinical. Table 3 shows the sex and ethnicity of the students referred in each course section. There appears to be an excess of males referred in the early years, particularly in the UG course (which admits 39% males overall, see Table 1), and to a lesser extent in the GEM course (59% males overall). In the clinical course, the proportion of males referred again exceeds the overall balance of 44%. These data are slightly skewed by students with multiple forms; 15 males had more than one form, 51 in total, compared to seven females with a total of 17 forms. Nevertheless, there are proportionately more forms issued to males than females. With regard to ethnicity, there may again be a slight skew as a result of multiple referrals, but there does seem to be a tendency for non-White students to be over-represented compared to the overall student population.

**Table 3:** **Sex and ethnicity of students referred (number of forms)**

	UG pre-clinical (5 semesters)	GEM pre-clinical (3 semesters)	Combined clinical (5 semesters)	Total
Males	48	11	65	124
Females	22	3	40	65
% Males	69	79	62	66
White	33	3	58	94
Non-white	30	11	40	81
Undeclared	7	0	7	14
% Non-white *	43	79	38	43

* Actual% Non-white could be slightly different because of students of undeclared ethnicity.

### Origins of the referrals

As mentioned, the names recorded in the database of the people submitting Concerns forms were replaced by a code for their status, in terms of teaching/non-teaching staff and various other occupational groups. Table 4 shows the origins of the referrals condensed into just four categories: clinical teachers & staff, non-clinical teachers & technical staff, faculty and administrative staff, and all others (which includes student peers, personal GPs, and Hall Wardens). Clearly over 50% of the Concerns raised originate with clinically-based teachers & staff, compared to ~10% raised by non-clinical teachers and technical staff. The remainder are shared equally between Faculty/administrative staff and the wider group of ‘others’. As would be expected, clinical staff are more likely to be the originators of the concern in the clinical course. (A more detailed breakdown is shown in Additional file 2).

**Table 4:** **Originators of the Concerns form, condensed categories**

	UG pre-clinical (5 semesters)	GEM pre-clinical (3 semesters)	Combined clinical (5 semesters)	Total (%)
Clinical teachers & staff	11	1	86	98 (52)
Non-Clinical teachers & staff	19	0	1	20 (11)
Faculty & administrative staff	11	10	16	37 (19)
All others	29	3	2	34 (18)
Totals	70	14	105	189

### Categorisation of information used by originators

As noted above, the current Concern form has four sections, and advice on what constitutes unprofessional behaviour, but no definitive guidance on the use of the sections. During data coding for the audit, it became clear that there was variation in the way the forms had been completed, eg some originators had used the ‘Other’ heading for inappropriate behaviour. Others had completed the form on paper, rather than electronically, and used more than one section perhaps because of lack of space rather than a deliberate choice of category. In coding the data, shown in Table 5, allowance therefore had to be made for this by adding a ‘combination of categories’. Additionally, Hall Wardens had referred by letter rather than by using a form, and these are shown separately. Inappropriate attitude or behaviour was clearly the most widely used category.

**Table 5:** **Categories used in the completion of the Concerns forms**

	UG pre-clinical (5 semesters)	GEM pre-clinical (3 semesters)	Combined clinical (5 semesters)	Total
Inappropriate attitude or behaviour	49	11	69	129
Serious misconduct	0	2	0	2
Health	4	0	12	16
Other	4	0	16	20
Combination of categories	2	1	7	10
Letters *	11	0	1	12
Totals	70	14	105	189

* These were sent by Hall Wardens who had not used the standard forms.

### Action taken in response to Concern forms

When Concern forms are received by the course office, they are logged by the relevant staff member and then reviewed in conjunction with a senior person, normally a Senior Tutor or Clinical Sub-Dean. The action taken subsequently is determined according to the type of problem raised. This could range from a simple advisory email to a formal Fitness to Practice hearing and review.

Table 6 summarises the action taken in response to these 189 forms. As would be expected, the Senior Tutors saw the majority of students from the early years of the course, whereas the Clinical Sub-Deans were heavily involved with students in the later years. Those cases referred to the Faculty Secretary reflect serious concerns about a student’s fitness to practise and in some cases proceeded to a formal hearing.

**Table 6:** **Onward referral of the Concerns forms**

	UG pre-clinical (5 semesters)	GEM pre-clinical (3 semesters)	Combined clinical (5 semesters)	Total (%)
Advice	3	3	0	6 (3)
Referred to Senior Tutor	48	0	1	49 (26)
Referred to Clinical Sub-Dean	2	0	88	90 (48)
Referred to Medical Course Manager	10	0	3	13 (7)
Referred to Associate Dean for Medical Education	7	4	8	19 (10)
Referred to Faculty Secretary	0	7	4	11 (6)
Not known *	0	0	1	1 (−)
Totals (row%)	70 (37)	14 (7)	105 (56)	189

* Original Form could not be located.

### Mapping of the concerns to the GMC areas of professional behaviour

**Figure 1:**
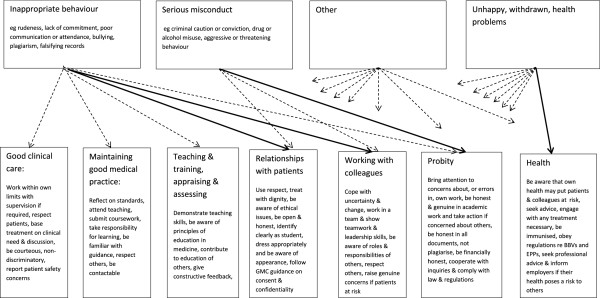
**Mapping of categories of Concerns to areas of GMC professional values & fitness to practise.**

Figure 1 illustrates the way in which we attempted to map the four general areas of the blank Concerns form to the seven categories of professional behaviour detailed in the GMC documentation [7]. Solid lines represent primary linkages – very clear relationships between the two groups – and dashed lines represent secondary linkages – more variable or debatable relationships.

We then highlighted key free-text information within each completed Concern form and allocated it to one or more of the seven GMC categories. Tables 7 and 8 illustrates the most common types of information seen in the forms and the subsequent mapping, with the numerical distribution of the concerns in these areas – they are not mutually exclusive and many forms were mapped to two or more GMC areas. Overall, 277 mapping points were identified.

**Figure 2:**
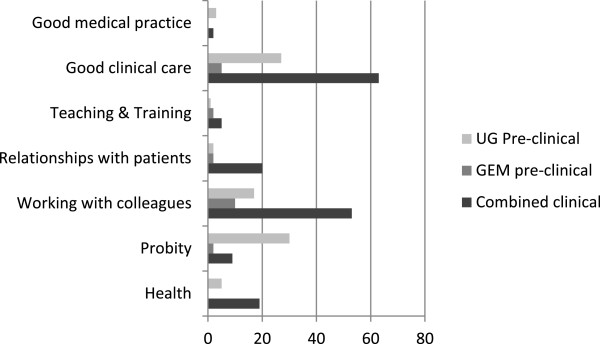
**Distribution of mapping points against GMC categories of professional behaviour.**

Finally we counted the GMC-mapped categories for each academic year group, and these are shown in Figure 2. This shows clearly the increased complaints during the clinical years related to Good Medical Practice and Working with Colleagues, and also that more complaints relating to Probity are logged during the early, non-clinical, parts of the course.

**Table 7:** **Mapping exercise of Concerns to GMC categories of professional behaviour**

GMC category	Number of Concerns forms mapped to each category	Examples of behaviour mapped to each category
Good clinical care	5	Inappropriate comments made to a patient in front of others
		Inappropriate advice to a patient
		Giving other students inappropriate advice about clinical care
		Illegible writing
		Failing to listen to patients’ opinion
		Failing to contribute to patient care
Maintaining good medical practice	95	Absence from teaching with notice or prior permission
		Failure to follow the timetable and/or get assignments signed off
		General lack of commitment to teaching & learning activities and/or tutor meetings
		Failure to engage with research project, poor note-keeping and general disorganisation
		Ignoring emails or other contacts from teaching or administrative staff
Teaching & Training	8	Disruptive behaviour in group teaching sessions
		Dismissive or arrogant behaviour to other individuals during teaching
Relationships with patients	24	Rudeness to colleague in presence of simulated patient
		Making a patient feel uncomfortable during examination
		Inconveniencing patients by not attending and not appreciating the problems caused
		Not respecting professional boundaries (deciding to visit a patient at home)
		Abrupt and non-empathetic manner with patients
Relationships with colleagues	80	Rude or aggressive to fellow students or to staff, with confrontational, intimidating or arrogant behaviour
		Making fun of others inappropriately
		Using offensive language during teaching sessions
		Lack of engagement with clinical teams, disrespect, lack of insight into behaviour
		Poor body language, inattention, disinterest and casual behaviour
Probity	41	Plagiarism or fabrication in written work
		Failing to obey rules & regulations, particularly in Halls of Residence
		Giving false identification when challenged
		Drunk & disorderly behaviour in Halls, noise disturbance
		Asking another student to sign them in for teaching, or signing another in themselves
		Arrest or criminal offence
		Writing rude/inappropriate comments on exam script
Health	24	Work or attendance affected by health disorders such as depression
		Student failing to appreciate the effects of poor health on performance and seek support
		Ongoing illness which may affect future ability to function as a doctor
		Adversely affected by serious personal or relationship problems

**Table 8:** **Summary of results from the mapping exercise, by academic year group**

	Number of times that the category was mapped for each stage of the course			
	UG pre-clinical (5 semesters)	GEM pre-clinical (3 semesters)	Combined clinical (5 semesters)	Total (%)
Good medical practice	3	0	2	5 (2)
Good clinical care	27	5	63	95 (34)
Teaching & Training	1	2	5	8 (3)
Relationships with patients	2	2	20	24 (9)
Working with colleagues	17	10	53	80 (29)
Probity	30	2	9	41 (15)
Health	5	0	19	24 (9)
Total	85	21	171	277

### The ‘repeat offenders’

As noted above, 22 students had more than one Concerns form logged, so the database was examined to determine whether these students had been referred for similar reasons. For reasons of confidentiality we are unable to list these Concerns by individual student. However, we are able to say that the majority of repeat forms for any one student do indeed relate to similar problems, particularly in terms of unprofessional behaviour or attitude.

## Discussion

This audit has explored the use of a standardised form to record complaints about student behaviour, and explored its use in a variety of circumstances. To our knowledge, this is the first report of such data collected within a UK medical school. The study also examines the potential relationships between our categories of Concerns and the GMC definitions of Professional Behaviour.

We do not propose to discuss the numerical results in detail because they are generated from a small sample and may not be reliable or generalizable. However, the finding that males and ethnic minority students may be at greater risk of adverse incidents needs to be re-examined in the future. The slight excess of UG over GEM students may simply reflect their younger age and less mature behaviour. The increase in referrals during the clinical course probably results from increased scrutiny of behaviour by clinical staff. In terms of the overall student body, the referral rate is low.

The Concerns form has proved to be usable in a wide range of circumstances, and by the full range of people who come into contact with medical students. It is clearly of use in the clinical scenario, where it is important in providing a valid and defensible means of reporting unprofessional behaviour. Many of the complaints described poor behaviour and language towards patients or fellow students, or unsatisfactory team-work, both highly important with respect to patient care and safety. Indeed, a recent questionnaire survey from Manchester has suggested that members of the public may judge student behaviour even more harshly than do doctors, who are also less lenient than students themselves [8]. Issues relating to probity were more common in the early, non-clinical course, and largely centred on plagiarism in written work, on lack of respect for rules (such as smoking in Halls of Residence, where it is forbidden), and falsifying signatures. A recent survey in Dundee Medical school suggests that students may be less concerned about these issues then their Faculty staff [9]. It has been suggested that the common use of social networking amongst students may be blurring the distinction between acceptable and non-acceptable behaviour, and between private and professional life, underlining the need for clear guidance and rules [10]. A more recent survey has underlined a lack of awareness amongst some doctors and students [11]. Nottingham now provides departmental advice about the use of social networking [12]. The GMC also provides specific advice for doctors [13] and more informal advice aimed at students [14].

The form was also used on a number of occasions to record anxieties about a student’s health, especially mental health problems such as recurrent depression or disabling anxiety which were affecting performance. A recent survey in the US has pointed to a positive link between students’ mental health and their professional behaviour [15], demonstrating the value of identifying students who may need extra help and support. Current documentation from the UK Medical Schools Council and GMC stress that mental health problems should be dealt with primarily through support and appropriate care, but acknowledge that such illness may also affect professional behaviour [16]. We therefore included health issues as a category for concern, to ensure that affected students receive appropriate help.

Our algorithm provided a satisfactory means of escalating the response to the Concern form in an appropriate way. The majority were dealt with by Faculty pastoral care staff, but 19 required personal attention by the Associate Dean for Medical Education and a further 11 were appraised by the Dean and Faculty Secretary, reflecting very serious concerns about the students’ fitness to practise. It would be an interesting exercise in future to use the algorithm in the opposite direction, by reviewing all Fitness to Practice cases to see whether they had originally been associated with Concerns forms. This would help to confirm whether or not the system was operating efficiently.

Although the form was used successfully to record a wide variety of Concerns, the guidance provided to the originators about which category to use was sometimes insufficient, and of course some complaints covered more than one area. We found therefore that the categories provided were not always used consistently. Another minor problem affecting audit was that the question about the context of the incident was not always answered as we wished; some people wrote ‘medical student’ rather than their own status, and a number put their name but not their professional status or job. Clearly some improvements are needed now that the form has been operational for some time.

Students are provided with copies of the GMC’s documentation on professional attitudes and behaviour, and our mapping exercise suggests that these seven categories can be used to define the Concerns more accurately. This would enable future audit to focus more clearly on the aspects of unprofessional behaviour that are the most frequent or serious, and therefore need to be given more prominence during teaching.

As a result of the audit we formulated several recommendations for change, which are currently being considered:

1. Redesigning the form with check boxes which align with the GMC categories, including brief explanatory text as necessary, would be helpful. Originators could then indicate more precisely which categories of behaviour were relevant as well as adding free-text information to describe the incident more fully as necessary.
2. It would be useful for future analysis if a field for the sex of each student was added. (Ethnicity cannot be added routinely since this information is only held on the central University databases and not departmentally).
3. An additional field should be used, after the name of the referring person, for their status/job title at the time of the incident. At a minimum this could be the four overall groupings shown in Table  4. Alternatively the more detailed groupings in Additional file 2 could be used, and then the database operator could add the overall grouping level.
4. The receipt of two or more forms for one student, especially if describing similar incidents, may deserve close attention.
5. Other departmental work which has shown that failure to attend to immunisation requirements (such as Hepatitis B) may be a marker of a potentially struggling student [17]. This failure is, in itself, unprofessional behaviour, so we are considering the automatic issue of a Concern form for such students unless they have a valid reason.

### Strengths and limitations

These data have been collected routinely from an open, transparent system, which is handled systematically and is therefore as robust as it can be. However, the data constitute a relatively small sample, having been collected over three years from the start-up of a new system, and in only one medical school. We cannot therefore claim that our results are generalizable to other schools, with a different intake and perhaps different levels of teaching about professional behaviour, but the overall concept is relevant.

### Further audit and research

We are not aware of other UK medical schools’ policy and procedure for dealing with unprofessional behaviour on a practical basis, and hope that some will now come forward to compare and contrast their systems and experience.

## Conclusions

A simple form, freely available, but designed to prevent frivolous or malicious use, has provided valuable data on unprofessional behaviour and the responses elicited. Some parts of the form require improvements, and these are underway to provide more efficient use, audit and review in future.

## Authors’ information

JY was a Research Fellow in Medical Education at Nottingham from 2003–2013 and has written and co-authored a number of papers on medical students’ progress and failure to thrive.

## Electronic supplementary material

Additional file 1: **Copy of the Concerns form and associated documentation in use at the time when the information was recorded and collected.** (PDF 184 KB)

Additional file 2: **Table 2.1: Detailed information about the originators of the Concerns forms.** (PDF 19 KB)
