# Supplementary material for: “Concerns” about medical students’ adverse behaviour and attitude: an audit of practice at Nottingham, with mapping to GMC guidance
Source: BMC Med Educ. 2014 Sep 20;14:196. doi: 10.1186/1472-6920-14-196 (PMC4189166; doi:10.1186/1472-6920-14-196)
Supplement: Supplementary file 1 — Additional file 1: Copy of the Concerns form and associated documentation in use at the time when the information was recorded and collected. (PDF 184 KB) [file 12909_2014_1030_MOESM1_ESM.pdf]

Additional File 1: The ‘Concerns’ form as it was used during the period of this audit.

Faculty of Medicine and Health Sciences
University of Nottingham
Medical School

Guidance on use of the medical student ‘Concern Form’

Introduction

1. The development of professional behaviour is a vital part of undergraduate medical education. It is also imperative that where inappropriate behaviour exists it must be identified so that the safety and care of patients is not jeopardised. The concern form is designed to provide an effective means of communicating concerns about medical students to the Medical School so that appropriate action may be taken to address them. It should be remembered that all medical students sign a code of conduct on entry to the medical course which describes fully the attitudes and behaviour which are expected of them.
2. The procedure is also intended to help identify medical students experiencing personal or health problems so that support can be put in place.
3. The form may be used by clinical staff responsible for supervising medical students, by other academic staff, by other health professionals with whom the student might come in contact or by patients and members of the public.
4. The form may also be used by medical students who have concerns about a fellow medical student. Indeed, medical students have a duty to report dangerous, abusive, discriminatory, dishonest or exploitative behaviour or practise.
5. This procedure accompanies the ‘Whistleblowing’ code of practice which gives guidance on how medical students should raise concerns about behaviour or practice in the University or the NHS which does not involve a medical student. The ‘Whistleblowing’ code of practice and the ‘Concern Form’ are designed to complement each other.

Examples of areas where a concerns form is appropriate

- Health Concern
- Alcohol/substance misuse
- Rudeness to patients, staff, colleagues
- Lack of commitment
- Inappropriate discrimination
- Inappropriate behaviour
- Inappropriate language
- Poor/lack of appropriate communication including inappropriate response to email communications from the Faculty
- Bullying or harassment
- Poor attendance
- Inappropriate conduct in teaching sessions
- Plagiarism or cheating
- Falsifying signoffs, for example attendance records at taught sessions

Note

1. A concern commonly results in pastoral care and guidance being provided. Not all concerns are expected to result in a disciplinary outcome.
2. Concerns raised anonymously will not normally be considered.
3. All concerns will be treated in confidence but we cannot guarantee that the identity of the person raising the concern will not be revealed. In the event of a concern about a student proceeding to the fitness to practise committee, only in exceptional circumstances will their identity **not** be disclosed.
4. Raising concerns maliciously, recklessly or irresponsibly will be considered to contravene the Nottingham University Code of Conduct for Medical Students and may result in a FTP referral in itself.

Action

1. Following receipt of a concern form, the Student Support and Development Administrator, Medical Course Office, will, as soon as possible either, refer the case to an appropriate Senior Tutor or Clinical Sub-Dean for investigation, or respond directly to the student for minor offences, such as non attendance. The student, about whom the allegation is made, will be interviewed if appropriate and their record reviewed.

2. Should the student not receive a standard warning email from the Student Development Administrator, upon investigation, the Senior Tutor or Clinical Sub-Dean will decide on one the following:
    - No action is warranted;
    - A warning should be given and/or appropriate support organised, for example referral to one or more of the following:
        - The University Counselling Service
        - Occupational Health
        - General Practice
        - The Academic Progress Committee
    - The issue is sufficiently serious to be referred to the Associate Dean for Medical Education.

    The student will be informed of the outcome of the investigation within 10 working days of the referral.

3. The Associate Dean for Medical Education will decide whether more serious cases should be referred to an appropriate committee or agency for consideration. Depending on the nature of the allegation and whether there have been previous expressions of concern, the student may be referred, for example to one or more of the following:
    - The Academic Offences Committee
    - The Senate Disciplinary Committee
    - The Fitness to Practise procedure.
    - Any other appropriate University procedure, for example such as to comply with the Dignity (harassment) Policy.
    - An external body such as an NHS Trust or, if criminal activity is suspected, the police.

    The student will be informed of this within 5 working days.

4. Guidance will be provided by the Curriculum Policy Group on the application of these procedures to ensure consistency, fairness and proportionality.

Support and Follow-Up

Where appropriate, the Senior Tutor/Clinical Sub Dean or their nominee will agree a support plan to help the student which may involve the Student Support Office, Academic Services, a Senior Tutor or Clinical Sub-Dean and/or medical support. A student may be required to attend remediation.

The staff member should ensure that follow-up continues until both the student and staff member agree that support is no longer needed. This decision will be documented and a record held in the student file.

October 2010

Approved by: Curriculum Policy Group, Medical School
Date For Review: April 2011

The University of Nottingham

Medical School
Notification of Concern about a Medical Student

**Student Name:**<br>**(block capitals)**	**Year of Study:**
**Please state nature of concern:**<br><br>1. Student unhappy/withdrawn/has health problems<br><br>2. Inappropriate attitudes or behaviour (for examples see 'guidelines on use of the medical student concern form')<br><br>3. Serious misconduct (e.g. a criminal conviction or caution/ drug or alcohol misuse/ aggressive or threatening behaviour)<br><br>4. Other	
**Report from:**<br>**(block capitals)**<br>**Address:**<br><br>**Telephone number:**	**Date:**<br><br><br><br>**Email:**

**Context in which this student has come to your attention, e.g. clinical teacher, personal tutor, medical student:**
**Signature** (If sent electronically a paper copy with signature should follow)
**This form may be kept on file indefinitely and could be used as evidence in a Fitness to Practice investigation.**
**Please return to: The Student Support and Development Administrator, Medical Course Office, B Floor, Medical School, QMC or email** studentconcerns@nottingham.ac.uk **For GEM students on the pre clinical course, please send these to the School Administrative Officer, School of GEM & Health, The Medical School, Royal Derby Hospital, Derby.**
