# Supplementary material for: “Concerns” about medical students’ adverse behaviour and attitude: an audit of practice at Nottingham, with mapping to GMC guidance
Source: BMC Med Educ. 2014 Sep 20;14:196. doi: 10.1186/1472-6920-14-196 (PMC4189166; doi:10.1186/1472-6920-14-196)
Supplement: Supplementary file 2 — Additional file 2: Table 2.1: Detailed information about the originators of the Concerns forms. (PDF 19 KB) [file 12909_2014_1030_MOESM2_ESM.pdf]

Additional File 2

Table 2.1 shows the origins of the forms received. Not surprisingly, Concerns lodged during the early course originated largely with non-clinical staff, including 11 from Hall Wardens relating to Year 1 students, who were the most likely to be living in Halls. Concerns relating to clinical course students were more likely to originate from clinical teachers and other health professionals, as might be expected. Quite a number throughout the course came from the combined group of Faculty administrators and Undergraduate course coordinators, and also from the Senior Faculty staff group, which included Senior Tutors and Clinical Sub-Deans.

Table 2.1: Originators of the Concerns forms, detailed categories

	Year 1	Year 2	Hons Year 3	GEM 1	GEM 2	CP1	CP2	CP3	Total
Clinical teachers		6	5		1	7	34	22	75
Clinical educators/technical staff						5	5	10	20
Nurses/other health professionals							2	1	3
Non-clinical teachers		1	2				1		4
Non-clinical educators/technical staff	4	6	6						16
Senior faculty staff/CSDs		7	1	6	3	2		3	22
Faculty Admin staff/UG co-ordinators		1	2		1		7	4	15
Fellow students	6		1		3				10
Personal GP/health centre staff	3	6						1	10
Patients/members of public		1							1
Hall Warden	11		1			1			13
Totals	24	28	18	6	8	15	49	41	189
